# Dynamics of sex ratio and female unmatedness under haplodiploidy

**DOI:** 10.1002/ece3.1045

**Published:** 2014-04-02

**Authors:** Andy Gardner

**Affiliations:** School of Biology, University of St AndrewsDyers Brae, St Andrews, KY16 9TH, U.K.

**Keywords:** Arrhenotoky, ecology, evolution, oscillation, sex allocation, virginity

## Abstract

Haplodiploid sex determination allows unmated females to produce sons. Consequently, a scarcity of males may lead to a significant proportion of females remaining unmated, which may in turn give rise to a surfeit of males in the following generation. Stable oscillation of the sex ratio has been predicted by classic models, and it remains a puzzle as to why this is not observed in natural populations. Here, I investigate the dynamics of sex allocation over ecological and evolutionary timescales to assess the potential for sustained oscillation. I find that, whilst stable oscillation of the sex ratio is possible, the scope for such dynamical behavior is reduced if sex allocation strategies are evolutionary labile, especially if mated females may facultatively adjust their sex allocation according to the present availability of mating partners. My model, taken together with empirical estimates of female unmatedness in haplodiploid taxa, suggests that sustained oscillation of the sex ratio is implausible in natural populations. However, this phenomenon may be relevant to artificially introduced biological control agents.

## Introduction

Around 20% of all animal species employ haplodiploid sex determination (Crozier and Pamilo 1996). In such species, whilst females are produced sexually, deriving from eggs that have been fertilized by sperm, males are produced asexually, deriving from unfertilized eggs, in a process termed “arrhenotoky”. Thus, haplodiploidy allows unmated females to produce offspring, of whom all are male.

Nearly a century ago, the insect ecologist C. B. Williams (1917) highlighted the potential for arrhenotoky to drive oscillation of the sex ratio. Specifically, a scarcity of males in one generation may lead to a significant proportion of females being unmated, which in turn leads to a surfeit of males in the next generation. Fifty years later, W. D. Hamilton (1967) provided an analytical treatment, reporting that this would lead to sustained oscillation of the sex ratio, provided *k* < (1−*e*)/*e* where *k* is the number of females than can be mated by each male and *e* is the sex ratio (proportion male) among the offspring of mated females. Such sustained oscillation obtains even when males are highly fecund (large *k*), provided that the sex allocation of mated females is sufficiently female biased (low *e*).

It remains a puzzle as to why such sustained oscillations are not, in fact, observed in the natural world. One possibility is that Williams' and Hamilton's predictions are artifacts of artificial model assumptions. For example, both authors treated the sex allocation of a mated female as a fixed parameter and did not consider its evolutionary dynamics. However, sex allocation does evolve by natural selection, and the presence of unmated females who are constrained to produce only sons favors mated females to allocate more resources to daughters (Godfray and Grafen 1988; Godfray 1990; West 2009). Consequently, the sex allocation of mated females is expected to co-evolve with the extent of female unmatedness, making some regions of Hamilton's parameter space more evolutionarily plausible than others. Thus, the true scope for female unmatedness to drive stable oscillation of the sex ratio under haplodiploidy is unclear.

Here, I extend Williams' and Hamilton's analyses by investigating evolutionary change in sex allocation and its impact on the ecological dynamics of the sex ratio and female unmatedness. First, I consider that mated females employ a fixed sex allocation strategy that is evolutionarily optimized according to the intergenerational average availability of mating partners. This is consistent with Hamilton's ecological model and clarifies the parameter range over which stable oscillation obtains. Second, I consider that mated females are able to facultatively adjust their sex allocation according to current availability of mating partners. This requires an extension of Hamilton's ecological model and further clarifies the parameter range over which stable oscillation obtains.

## Models and Results

### Obligate sex allocation

Following Hamilton (1967), I consider an infinite, haplodiploid population. Mating occurs as follows: females and males mate at random, with females leaving the mating pool upon their first mating and males leaving the mating pool upon their *k*th mating, such that mating ends when one or both of the sexes is no longer present in the mating pool. Mated females produce offspring with sex ratio *e,* and unmated females produce offspring with sex ratio 1, with all females producing the same number of offspring.

Thus, if the sex ratio in any generation is *z*, the proportion of females being mated in that generation is *m* = (*zk*)/(1−*z*) if *z* ≤ 1/(1 + *k*) and *m* = 1 if *z* > 1/(1 + *k*), and the sex ratio in the following generation is *z*′ = *m e* + 1−*m*, or:


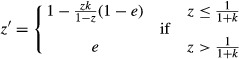
(1)

**Figure 1:**
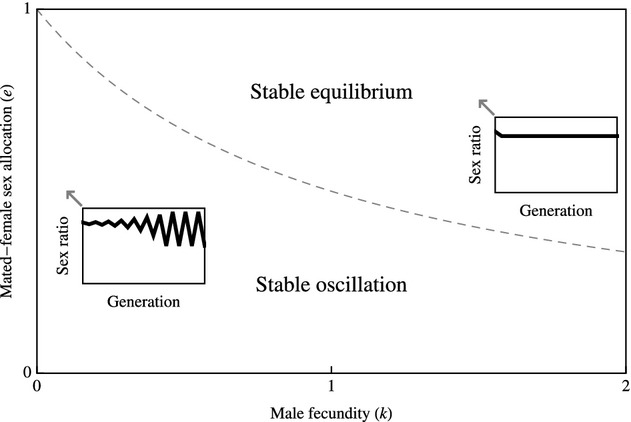
Hamilton's model. Stable oscillation of the sex ratio between *z* = *e* and *z* = 1−*ek* obtains when *k* < (1−*e*)/*e*, and a stable equilibrium at z* = ½(2 + *k*(1−*e*)−(*k*(1−*e*)(4 + *k*(1−*e*)))^½^) obtains when *k* ≥ (1−*e*)/*e*. Insets illustrate the scenarios indicated by arrows (*k* = 0.10 & *e* = 0.50 and *k* = 1.50 & *e* = 0.75).

**Figure 2:**
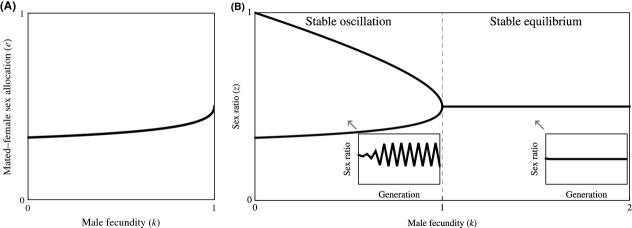
Obligate sex allocation. (A) Natural selection favors mated females to exhibit female-biased sex allocation *e* = (3 + *k*−((9−*k*)( 1−*k*))^½^)/(8*k*) when *k* ≤ 1 and unbiased sex allocation *e* = ½ when *k* ≥ 1. (B) This leads to stable oscillation of the sex ratio between *z* = (3 + *k*−((9−*k*)(1−*k*))^½^)/(8*k*) and *z* = (5−*k* + ((9−*k*)( 1−*k*))^½^)/8 when *k* < 1, and a stable equilibrium at z* = ½ when *k* ≥ 1. Insets illustrate the scenarios indicated by arrows (*k* = 0.50 and *k* = 1.50).

As Hamilton reported, this leads to stable oscillation in sex ratio between *z* = *e* and *z* = 1−*ek*, either side of an unstable equilibrium at z* = ½(2 + *k*(1−*e*)−(*k*(1−*e*)(4 + *k*(1−*e*)))^½^), if *k* < (1−*e*)/*e* (see Appendix A1 for derivation; Fig. 1). However, the sex allocation of mated females is not arbitrary. Rather, this trait is expected to be under strong selection to counter the male bias introduced by the reproduction of unmated females (Godfray and Grafen 1988; Godfray 1990; West 2009). If mated females adopt a sex allocation *e* that is not facultatively adjusted according to mate availability, then natural selection leads this to converge upon:


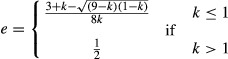
(2)

(see Appendix A1 for derivation; Fig. 2A). This result extends that of Godfray (1990), who assumed a fixed rate of female unmatedness, to a scenario in which unmatedness fluctuates over generations. Whilst Godfray (1990) showed that mated females are favored to adopt a female-biased sex allocation that exactly negates the male bias introduced by female unmatedness, here, I find that mated females are forced to overcompensate when male fecundity is limiting and undercompensate when male fecundity is not limiting.

This development is compatible with Hamilton's model of the ecological dynamics of the sex ratio, but reduces its parameterization. In particular, whereas Hamilton's model is governed by two parameters – male fecundity (*k*) and mated-female sex allocation (*e*) – I have expressed the latter in terms of the former, so that the model is solely governed by the male-fecundity (*k*) parameter. Returning to Hamilton's original condition *k* < (1−*e*)/*e* for stable oscillation, and substituting *e* for the solution given in equation (2), the condition for stable oscillation becomes *k* < 1. In other words, stable oscillation obtains only if each male can, on average, mate with fewer than one female. In this case, the sex ratio stably oscillates between *z* = (3 + *k*−((9−*k*)(1−*k*))^½^)/(8*k*) and *z* = (5−k+((9−*k*)(1−*k*))^½^)/8; that is, the sex ratio is alternately female biased and male biased in successive generations, and on average, it is male biased (Fig. 2B). Otherwise, if *k* ≥ 1, all females are guaranteed of being mated when the sex ratio is *z* = ½, and the adoption of the sex allocation strategy *e* = ½ ensures that the sex ratio z* = ½ remains stable (Fig. 2B; cf Williams 1917).

### Facultative sex allocation

**Figure 3:**
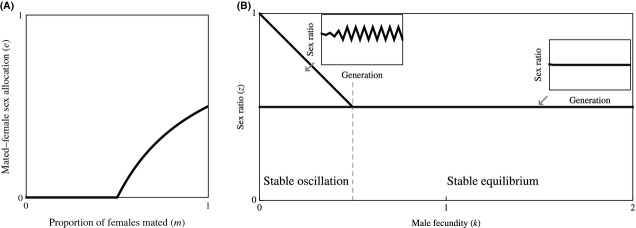
Facultative sex allocation. (A) Natural selection favors mated females to exhibit female-biased sex allocation *e*(*m*) = 0 when *m* ≤ ½ and *e*(*m*) = (2 *m*−1)/(2 *m*) when *m* ≥ ½, where *m* is the proportion of females that are mated in this generation. (B) This leads to stable oscillation of the sex ratio between *z* = ½ and *z* = 1−*k* when *k* < ½, and a stable equilibrium at z* = ½ when *k* ≥ ½. Insets illustrate the scenarios indicated by arrows (*k* = 0.25 and *k* = 1.50).

I now assume that mated females produce offspring with sex ratio *e*(*m*), which is facultatively adjusted according to the proportion *m* of females that are mated in their generation. Natural selection favors the sex allocation strategy:


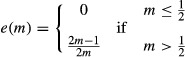
(3)

(see Appendix A1 for details; Fig. 3A). This is Godfray's (1990) result, and here, I have shown that it extends to a more complicated scenario than the one he considered. Specifically, whilst Godfray (1990) assumed that the proportion of mated females remains fixed over successive generations, I have considered that it may fluctuate between generations. Crucially, I have considered that females respond facultatively to this fluctuation, rather than adopting a fixed sex allocation that optimizes with respect to average mate availability (as was done in the previous section).

This development is not compatible with Hamilton's model of the ecological dynamics of the sex ratio, which assumes that mated females adopt the same sex allocation in all generations, irrespective of the current availability of males. Consequently, I extend Hamilton's model to consider intergeneration variation in the sex allocation of mated females. As in Hamilton's model, if the sex ratio in any generation is *z*, then the proportion of females being mated in that generation is *m* = (*zk*)/(1−*z*) if *z* ≤ 1/(1 + *k*) and *m* = 1 if *z* > 1/(1 + *k*), and the sex ratio in the following generation is *z*′ = *m e*(*m*) + 1−*m*. Making the substitution given in equation (3) yields:


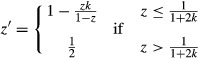
(4)

This leads to stable oscillation in sex ratio when *k* < ½. In this case, the sex ratio stably oscillates between *z* = ½ and *z* = 1−*k*; that is, the sex ratio is alternately unbiased and male biased in successive generations, and on average, it is male biased (Fig. 3B). Otherwise, if *k* ≥ ½, mated females are guaranteed to be able to set the population sex ratio in the next generation to *z* = ½ given that it is currently at *z* = ½, and hence, the sex ratio z* = ½ remains stable (Fig. 3B).

## Discussion

Building upon the numerical model of Williams (1917), Hamilton (1967) showed that female unmatedness could drive sustained ecological oscillation of sex ratio in haplodiploid populations. Here, I have assessed the impact that evolutionary optimization of the sex allocation behavior of mated females has upon such dynamics. Further to Hamilton's suggestion that sustained oscillation obtains even for arbitrarily large male fecundity, so long as mated-female sex allocation is sufficiently female biased, I have found that evolutionary optimization of mated-female sex allocation behavior places strict upper limits upon male fecundity, above which stable oscillation does not obtain.

First, I considered that mated females optimize their sex allocation behavior according to the average level of female unmatedness experienced over evolutionary timescales (obligate sex allocation). I found that stable oscillation in the sex ratio obtains only when each male can, on average, mate with fewer than one female. Otherwise, an even sex ratio guarantees that all females are mated, and this ensures that all females – adopting an even sex allocation strategy – can recover an even sex ratio in the subsequent generation. In the event that male fecundity is sufficiently low for stable oscillation in the sex ratio to obtain, mated females are favored to adopt a female-biased sex allocation strategy that undercompensates for the male bias introduced by unmated females in those generations in which there is a scarcity of males, and overcompensates in those generations in which there is a surfeit of males. The result is a sex ratio that oscillates between male bias and female bias in alternate generations, with an average male bias.

Second, I considered that mated females optimize their sex allocation behavior according to the present level of female unmatedness experienced in their generation (facultative sex allocation). I found that stable oscillation in the sex ratio obtains only when each male can, on average, mate with fewer than 0.5 females. Otherwise, an even sex ratio guarantees that at least half of all females are mated, and this ensures that all females – adopting a suitably female-biased sex allocation strategy – can recover an even sex ratio in the subsequent generation. In the event that male fecundity is sufficiently low for stable oscillation in the sex ratio to obtain, mated females are favored to adopt a female-biased sex allocation strategy that exactly compensates for any male bias owing to female unmatedness.

My obligate sex allocation analysis generalizes that of Godfray (1990), who considered optimization of mated-female sex allocation in a constant mating environment, without sex-ratio oscillation. Godfray (1990) found that mated females are favored to adopt a sex allocation of *e* = (2 *m*−1)/(2 *m*), where *m* is the frequency of mated females. In the context of fluctuations in rates of unmatedness, this becomes *e* ≈ ((2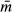 −1)/(2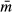 )) + (*σ*_*m*_^2^/(2
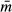
^3^)) where 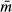 is the average and *σ*_*m*_^2^ the variance in the frequency of mated females (see Appendix A1 for details). My facultative sex allocation analysis yields a result identical to that given by Godfray (1990), but in a novel context. Specifically, if mated females employ obligate sex allocation in a constant mating environment (Godfray 1990), or facultative sex allocation in a variable mating environment (this paper), they are favored to adopt the strategy *e* = (2 *m*−1)/(2 *m*).

These developments build extra realism into Hamilton's (1967) model of sex-ratio dynamics. Usually, greater realism is achieved by adding parameters to models, making them more complex. However, greater realism has been achieved here by removing a parameter, by considering that sex allocation is an evolving variable. Other ways in which the model could be made more realistic include considering that the number of matings per male may be dependent upon the sex ratio, rather than taking a constant value, and allowing females imperfect information as to their mating environment. I leave these as avenues for future exploration.

Evolutionary optimization of sex allocation behavior greatly restricts the conditions under which stable oscillation may occur in natural populations. If mated females are obliged to employ a fixed sex allocation behavior, then even a small proportion of females remaining unmated may be sufficient to drive sustained oscillation in the sex ratio. But if mated females can facultatively adjust their sex allocation according to current male availability, then sustained oscillation requires that a majority of females remain unmated in every generation. Empirical estimates of the incidence of unmated (or otherwise “constrained”) females in haplodiploid taxa range from 0 to 30% and are usually <5% (West 2009), so there appears to be very little scope for sustained sex-ratio oscillation in natural populations. However, unmated females could plausibly outnumber mated females in artificially introduced biological control species (Rhainds 2010), especially at range frontiers and if dispersal is female biased (Heimpel and Asplen 2011). Haplodiploid taxa – especially parasitoid wasps – have been particularly favored for controlling agricultural pests (Waage and Hassell 1982). More generally, even transient oscillation may be long lasting over economically relevant timescales, making this an important factor in the design of biological control programs.

## Appendix A1: Dynamics of sex ratio and female unmatedness

### Obligate sex allocation

#### Ecological dynamics

Inspection of equation (1) suggests a candidate equilibrium at *z** = *e*, and this requires *e* ≥ 1/(1 + *k*). If *e* + *δ* ≥ 1/(1 + *k*), where *δ* is a vanishingly small quantity, then *z*′ = *e* when *z* = *e* + *δ*, so the equilibrium *z** = *e* is stable if *e* > 1/(1 + *k*) or, equivalently, *k* > (1−*e*)/*e*. A second candidate equilibrium is found by setting *z*′ = 1− (*zk*/(1−*z*))(1−*e*) = *z* and solving for *z* = *z** to yield *z** = ½(2 + *k*(1−*e*)−(*k*(1−*e*)(4 + *k*(1−*e*)))^½^). This assumes *z** ≤ 1/(1 + *k*), which is equivalent to *k* ≤ (1−*e*)/*e*. Setting *z* = *z** + *δ* yields *z*′ = *z**− (1/*z**)*δ* + O(*δ*^2^) which, as the magnitude of coefficient of *δ* is never less than unity, means that the equilibrium is unstable. Finally, if the sex ratio in any generation is *z* = *e*, then the proportion of females that are mated is *ek*/(1−*e*), and the sex ratio in the subsequent generation is *z*′ = 1−*ek*, which is sufficient for all females in that generation to be mated because *k* ≤ (1−*e*)/*e*. Hence, the sex ratio in the generation after that is *z*″ = *e*. Thus, the sex ratio oscillates between *e* and 1−*ek*.

#### Evolutionary dynamics

Sex allocation can be modeled by assigning every female an equal number of sons and daughters and then eliminating half of the offspring of each female according to her mating status and her sex allocation strategy (Taylor and Frank 1996). Thus, the probability that a focal female survives this culling is *m*(1−*x*), and the probability that a focal male survives this culling is *mx* + 1−*m*, where *m* is the probability that their mother is mated and *x* is the proportion of their mother's reproductive resources that is invested into sons in the event that she is mated. The population average female survival is *m*(1−*e*), and the population average male survival is *me* + 1−*m*. Hence, the relative fitness of a focal female is *W*_f_ = (*m*(1−*x*))/(*m*(1−*e*)) = (1−*x*)/(1−*e*), and the relative fitness of a focal male is *W*_m_ = (*mx* + 1−*m*)/(*me* + 1−*m*). Natural selection maximizes *W* = ∑_*m*_ *π*_*m*_(*c*_f_ *W*_f_ + *c*_m_ *W*_m_), where *π*_*m*_ is the proportion of generations in which the frequency of mated females is *m*, and *c*_f_ = 2/3 and *c*_m_ = 1/3 are the class reproductive values of females and males, respectively (Fisher 1930; Taylor 1996; Taylor and Frank 1996). That is, if a focal locus controls the sex allocation *e* of mated females, then, drawing a gene from this locus at random from the population and denoting its genic value by *g*, the condition for natural selection to favor an increase in *e* is d*W*/d*g* > 0, that is, ∑_*m*_ *π*_*m*_(*c*_f_ d*W*_f_/d*g*_f_ + *c*_m_ d*W*_m_/d*g*_m_) > 0. Here, d*W*_f_/d*g*_f_ = (∂*W*_f_/∂*x*) × (d*x*/d*G*) × (d*G*/d*g*_f_), where *G* is the genetic “breeding” value of the individual's mother for the sex allocation phenotype, d*x*/d*G* = 1 is the genotype–phenotype map and d*G*/d*g*_f_ = ¼ is the consanguinity of mother and daughter (Taylor and Frank 1996). Similarly, d*W*_m_/d*g*_m_ = (∂*W*_m_/∂*x*) × (d*x*/d*G*) × (d*G*/d*g*_m_), where d*G*/d*g*_m_ = ½ is the consanguinity of mother and son. This yields the condition ∑_*m*_ *π*_*m*_ (*m*/(*m e* + 1−*m*)) – (1/(1−*e*)) > 0. In general, this leads to a convergence stable value *e* ≈ ((2  −1)/(2 )) + (*σ*_*m*_^2^/(2)), where  is the average and *σ*_*m*_^2^ the variance in the proportion of females being mated (the approximation neglects higher orders of variance). However, of immediate interest is the scenario where the sex ratio oscillates between *e* and 1−*ek*, so that *m* takes values *m*_1_ = *ek*/(1−*e*) and *m*_2_ = 1 with equal probability. Thus, the condition for increase in *e* is (*m*_1_/(*m*_1_ *e* + 1−*m*_1_)) – (1/(1−*e*)) + (*m*_2_/(*m*_2_ *e* + 1−*m*_2_)) – (1/(1−*e*)) > 0, which yields an exact convergence stable value for *e*, given by equation (2).

### Facultative sex allocation

#### Evolutionary dynamics

The expected fitness of a juvenile female whose mother's generation involved a proportion *m** of females being mated is *w*_f_ = *m** (1−*x*), and the expected fitness of a juvenile male whose mother's generation experienced a proportion *m** of females being mated is *w*_m_ = *m** *x* + 1−*m**, where *x* is the proportion of their mother's reproductive resources that is invested into sons in the event that she was mated. Noting that the population average of *x* is  = *e*(*m**), the average fitness for females and males in such generations is  = *m**(1−*e*(*m**)) and  = *m** *e*(*m**) + 1−*m**, respectively. As before, the condition for natural selection to favor an increase in *e*(*m**) is ∑_*m*_ *π*_*m*_(*c*_f_ d*W*_f_/d*g*_f_ + *c*_m_ d*W*_m_/d*g*_m_) > 0, and as the gene is only active when *m* = *m**, this reduces to *π*_*m**_ (*c*_f_ d*W*_f_/d*g*_f_ + *c*_m_ d*W*_m_/d*g*_m_) > 0 where derivatives are evaluated at *m* = *m** and *x* = . This yields the condition –(1/(1−*e*(*m**))) + (*m**/(*m** *e*(*m**)+1−*m**)) > 0, such that the convergence stable sex allocation strategy for mated females is given by equation (3).

#### Ecological dynamics

Inspection of equation (4) suggests the candidate equilibrium *z** = ½, and this requires ½ ≥ 1/(1 + 2*k*), that is, *k* ≥ ½. If ½ + *δ* ≥ 1/(1 + *k*), where *δ* is a vanishingly small quantity, then *z*′ = ½ when *z* = ½ + *δ*, so the equilibrium *z** = ½ is stable if *k* > ½. A second candidate equilibrium is found by setting *z*′ = 1− (*zk*/(1−*z*)) = *z*, and solving for *z* = *z** to yield *z** = ½(2 + *k*−((4 + *k*)*k*)^½^). This assumes *z** ≤ 1/(1 + 2*k*), which is equivalent to *k* ≤ ½. Setting *z* = *z** + *δ* yields *z*′ = *z**−(4/(*k*^½^−(4 + *k*)^½^)^2^)*δ* + O(*δ*^2^) which, as the magnitude of the coefficient of *δ* is never less than unity, means that the equilibrium is unstable. Finally, if the sex ratio in any generation is *z* = ½, the proportion of females that are mated is *m* = *k*, and hence, the sex ratio in the subsequent generation is *z*′ = *m e*(*m*) + 1−*m* = 1−*k*. This leads to a proportion *m* = ((1−*k*)/*k*)*k* = 1−*k* of females being mated, and consequently, the sex ratio in the subsequent generation is *z*″ = *m e*(*m*) + 1−*m* = ½. Thus, the sex ratio oscillates between ½ and 1−*k*.
